# Gender-Specific Transmission of Depressive Symptoms in Chinese Families: A Cross-Lagged Panel Network Analysis Based on the China Family Panel Studies

**DOI:** 10.3390/bs15050672

**Published:** 2025-05-14

**Authors:** Xuanyu Zhang, Nan Fang, Rui Wang, Lixin Zhu, Dengdeng Zhang, Huina Teng, Boyu Qiu

**Affiliations:** 1 School of Mental Health, Guangzhou Medical University, Guangzhou 510180, China; 2 School of Stomatology, Guangzhou Medical University, Guangzhou 510180, China; 3 School of Health Management, Guangzhou Medical University, Guangzhou 510180, China

**Keywords:** cross-lagged panel network, depressive symptoms, familial transmission, gender difference

## Abstract

Depression is prevalent and may be transmitted within the family. However, whether and how gender influences the interaction of depressive symptoms between parents and adolescents remains largely unclear. The current study used a cross-lagged panel network (CLPN) analysis to examine the gender-specific transmission of depressive symptoms in representative Chinese families from the China Family Panel Studies. The participants included 1469 adolescents (48.3% girls) and their parents, with depressive symptoms assessed by the epidemiological studies depression scale in 2020 (T1; *M*_age_ = 13.80) and 2022 (T2; *M*_age_ = 15.62), respectively. The gender-specific CLPNs (i.e., boy–father, boy–mother, girl–father, and girl–mother CLPNs) showed that the “loneliness” at T1 repeatedly exhibited higher impacts on the other symptoms at T2 across networks. Furthermore, the symptoms of girls at T1 were more likely to influence their parents at T2, while the symptoms of boys at T2, especially the “sleep restlessness”, were susceptible to parental influence at T1. These findings provide deeper insights into the development of mental health policies, and future studies are needed to explore the mediating mechanisms of such transmission.

## 1. Introduction

Depression is a common mental disorder with familial aggregation (Branje et al., 2020; Lewis et al., 2017). According to the intergenerational transmission (Branje et al., 2020) and family systems theories (Cox & Paley, 2003), depression may be transmitted between parents and adolescents and mutually influence each other. Specifically, parental depression increases their children’s risk of developing depression through a variety of genetic and non-genetic factors. Children’s depression may lead to negative parenting styles (e.g., avoidance and punishment), mediating emotional dysregulation and depression in parents (Pardini, 2008). This reciprocal transmission of depression between parents and their children has been supported by a series of empirical studies (Schulz et al., 2021; Sifaki et al., 2021; Yan et al., 2021), which sheds light on interventions for the development of depression.

Most of the available studies investigating familial transmission relied on an aggregated measure of depression (W. Zhang et al., 2024). As a complex mental disorder, depression usually involves a combination of symptoms (Grimes et al., 2025). Therefore, treating depression as a latent entity limits the understanding of how its symptoms are transmitted between parents and children. To address this limitation, researchers administered network analysis (Epskamp et al., 2017) to examine the transmission of depressive symptoms within families (X. Gong et al., 2024; W. Zhang et al., 2024), providing more valuable insights into the underlying mechanisms. However, the influence of gender, which may play a crucial role in such transmission (Livings, 2021; Middeldorp et al., 2016), remains underexplored. In the current study, we used network analysis to explore the gender-specific transmission of depressive symptoms, thereby understanding the developmental mechanisms of mental disorders in families better.

### 1.1. Network Analysis to Explore the Transmission of Depressive Symptoms

Instead of treating mental disorders as latent entities, the network theory conceptualizes these disorders as interactions among a range of symptoms (Epskamp & Fried, 2018). A network is composed of nodes and edges, where nodes represent observable variables (e.g., various psychiatric symptoms across different populations), and edges denote the associations between these nodes (Epskamp et al., 2012). The importance and centrality of each node within the network are quantified by its expected influence (EI), which is the cumulative sum of all the positive and negative edges connected to the node. A node with a higher EI indicates that it has greater importance in the network (Robinaugh et al., 2016).

To further reveal the longitudinal dynamics among psychiatric symptoms, Rhemtulla et al. (2017) integrated network analysis with the cross-lagged panel model to construct the cross-lagged panel network (CLPN). The CLPN incorporates both the autoregressive and cross-lagged effects of the variables in the network (i.e., the coefficient for a node at T1 predicting itself or another node at T2 after controlling for all the other nodes at T1). The importance and centrality of each node within the CLPN are quantified by its in-expected influence (IEI) and out-expected influence (OEI). The IEI represents the proportion of variance in a given node at T2 that is explained by all the other nodes at T1, while the OEI reflects the impact of a given node at T1 on all the other nodes at T2 (Rhemtulla et al., 2017).

Nodes in the network can represent different types of psychiatric symptoms across populations, with nodes of the same type (e.g., various depressive symptoms forming a depressive community) or from the same population (e.g., symptoms in adolescents forming an adolescent community) gathering as a community. Bridging edges capture associations between nodes of different communities (Garabiles et al., 2019), and the cumulative weight of bridging edges connected to a given node is reflected in the bridge expected influence (BEI). A node with a higher BEI is more likely to activate other nodes in different communities (Heeren et al., 2018). BEI can also be applied to the CLPN, representing the influence of a given node at T1 on nodes in other communities at T2. Targeting highly central nodes in the CLPN provides an effective approach to treating mental disorders (Fried & Cramer, 2017).

Using nationally representative data from the China Family Panel Studies (CFPS), W. Zhang et al. (2024) applied the CLPN to explore the transmission of depressive symptoms within Chinese families. Specifically, they categorized the depressive symptoms of both parents and children living together into distinct community nodes, with longitudinal bridging edges reflecting the transmission of depressive symptoms between parents and their children. The results showed that the strongest bridging edge occurred from *child felt sad* (T1) to *mother felt depressed* (T2). Furthermore, both the father and the mother who felt depressed had the highest OEI scores, suggesting their important roles in influencing other depressive symptoms within the familial transmission network. Martin et al. (2023) similarly applied network modeling to investigate the transmission of depressive symptoms between parents and children in a UK population-based cohort. They found that both maternal and paternal anhedonia were highly associated with emotional difficulties in children. Although their studies advanced the understanding of depressive transmission in families, they did not explore the specific gender patterns in such a process.

### 1.2. Potential Gender Patterns in the Transmission of Depressive Symptoms

Gender, which is related to a series of physiological and psychological factors, may play an important role in the transmission of depressive symptoms within families (Hayward & Sanborn, 2002; Rose & Rudolph, 2006). For example, females typically have greater sensitivity of brain structures (e.g., amygdala) to emotional stimuli and fluctuations in hormone levels (e.g., estrogen) than males (NeuroLaunch Editorial Team, 2024). Such biological factors may contribute to the fact that girls often exhibit stronger emotional connections with their parents than boys, making girls more likely to influence and be influenced by their parents’ depressive symptoms. From a cultural perspective, especially in China, parents may have different expectations for their sons (e.g., hoping their sons to be independent and capable) and daughters (e.g., hoping their daughters to be obedient and modest). Different expectations may mediate different parenting styles, which, in turn, influence the transmission of depressive symptoms between parents and their children (Luo et al., 2013). Additionally, in some Chinese families with strong traditional values (Liu et al., 2021), sons tend to receive more parental expectations than daughters, which means that boys are more likely to be treated strictly or to be affected by parental negative symptoms during interactions.

Therefore, whether and how maternal and paternal psychiatric symptoms interact with psychiatric symptoms in female and male offspring is complex and remains a point of contention. Several empirical studies have tried to explore and highlight potential gender differences in the transmission of depression (Livings, 2021; Middeldorp et al., 2016; Schulz et al., 2021; Sifaki et al., 2021). Schulz et al. (2021) suggested that maternal, but not paternal, internalizing symptoms increased internalizing symptoms in adolescents. In contrast, Sifaki et al. (2021) argued that paternal psychological distress could also predict subsequent child emotional symptoms. Furthermore, Livings (2021) found that maternal depressive symptoms may have a greater impact on female than male offspring, whereas Middeldorp et al. (2016) found no such gender difference. However, these studies similarly relied on an aggregated measure of depression, which limits their ability to explore the specific symptom-level sources of gender differences.

Recently, X. Gong et al. (2024) administered the CLPN to examine the gender-specific transmission of depression in Chinese families. At the symptom level, they identified differences in the bridging edges between the boy–mother and girl–mother CLPNs, with the strongest connections from *boy felt worried* (T1) to *mother felt distractible* (T2), and from *girl felt worried* (T1) to *mother felt powerless* (T2). However, their study has certain limitations that restrict a comprehensive understanding of the gender-specific transmission of depressive symptoms. Firstly, they focused solely on maternal depressive symptoms, neglecting paternal symptoms. Secondly, they did not report the centrality indices (i.e., IEI, OEI, and BEI) of the boy–mother and girl–mother CLPNs. Last but not least, their sample was drawn from public elementary schools in a medium-sized city in northeast China, limiting its national representativeness.

At present, the prevalence of familial depression remains high (World Health Organization, 2017). Gender is considered to be a critical factor in influencing the interactive process of depressive symptoms in parents and children. Systematically elucidating the gender-specific transmission of depressive symptoms by CLPN in a nationally representative sample helps to (1) provide more valuable and detailed insights (i.e., based on the specific edges and centrality indices of the symptomatic transmission network) to disrupt the transmission of depressive symptoms early within families and (2) offer an experimental basis to further expand the framework of the intergenerational transmission and family systems theories, thereby improving the effectiveness of mental health policies and enhancing family-based practices.

### 1.3. Current Study

Using data from a large-scale longitudinal study in China—the CFPS (Xie & Hu, 2014), the current study aimed to explore the gender-specific transmission of depressive symptoms within families through network analysis. First, we separately constructed parent–adolescent CLPNs based on gender categorization (i.e., boy–father, boy–mother, girl–father, and girl–mother CLPNs) and verified the stability of these networks. Second, we calculated the centrality indices (i.e., OEI, IEI, and BEI) for each stable CLPN. Based on the existing evidence (X. Gong et al., 2024; W. Zhang et al., 2024), we hypothesized that each CLPN would exhibit a specific and relatively stable pattern of depressive transmission. Finally, we followed the guidelines for intergroup comparison of CLPNs (Rhemtulla et al., 2017) to indicate global (i.e., estimating the correlation of all edges between different networks) and local (i.e., comparing values of specific edge/centrality index in different networks) homogeneity and heterogeneity among these networks. Given the limited focus on gender-specific transmission in previous studies, we did not propose specific hypotheses regarding similarity or dissimilarity among these CLPNs. Instead, we aimed to explore the potential patterns that may provide insights into the controversial gender differences in the transmission of depression.

## 2. Methods

### 2.1. Data Collection Procedures

The CFPS is a longitudinal study investigating various aspects of Chinese family structure, economic conditions, population health, and social relationships. It recruited participants from 25 provinces across China, which account for nearly 95% of the national population distribution (Xie & Hu, 2014). All the procedures of the CFPS were approved by the ethical committee of Peking University, Beijing, China (approval number: IRB00001052-14010), and all the participants provided informed consent prior to participation. The present study used data from 2020 (T1) and 2022 (T2) of the CFPS (http://www.isss.pku.edu.cn/cfps, accessed on 15 November 2024) to explore the transmission of depression within the family. Participants were excluded if (1) they lost complete data on depressive symptoms at T1 or T2 and (2) adolescents and their parents did not live together at T1 or T2. Based on the above criteria, we excluded 1436 adolescents, thereby including 1469 adolescents (48.3% female; *M*_age_ = 13.80 years, ranging from 9 to 16 years at T1) and their parents in the final sample. Descriptive statistics for the demographic characteristics and depressive symptoms of the included participants are presented in Table 1 and Table 2, respectively.

### 2.2. Measurement

#### 2.2.1. Depressive Symptoms

In this study, the 8-item Center for Epidemiologic Studies Depression Scale (CES-D-8; Radloff, 1977) was used to assess the participants’ depressive symptoms, including D1 depressiveness (“I felt depressed.”), D2 effortfulness (“I felt everything I did was an effort.”), D3 restlessness (“My sleep was restless.”), D4 unhappiness (“I was happy.”, reverse scored item), D5 loneliness (“I felt lonely.”), D6 unenjoyment (“I enjoyed life.”, reverse scored item), D7 sadness (“I felt sad.”), and D8 felt life could not go on (“I felt my life could not go on.”). All the participants (including parents and children) self-reported each item on a 4-point scale, ranging from 1 = rarely or none of the time to 4 = most or all of the time. The CES-D-8 has shown good validity among Chinese people (Jiang et al., 2019; Rhemtulla et al., 2012). The Cronbach’s *α* for the CES-D-8 in the current sample was 0.76 at T1 and 0.77 at T2, indicating an acceptable internal consistency.

#### 2.2.2. Demographic Covariates

In this study, adolescents’ age, ethnicity, annual family income, and parental educational attainment were included as covariates in all the analyses, as these demographic variables may influence the transmission of depressive symptoms within the family (de Laat et al., 2018; X. Gong et al., 2024). The adolescents’ ethnicity was coded into two categorical variables: 1 = *Han* and 2 = *non-Han*, while parental educational attainment was categorized into four levels, ranging from 1 = *Primary school and below* to 4 = *Bachelor and above*. The other covariates remained in the continuous format to be controlled.

### 2.3. Statistical Analysis

All the data analyses were conducted on R (v4.4.1; R Core Team, 2024). First, the random forest imputation method in the *missForest* package (Stekhoven & Bühlmann, 2012) was used to handle missing data on covariates (i.e., adolescents’ age, ethnicity, annual family income, and parental educational attainment).

Second, we separately constructed parent–adolescent CLPNs based on gender categorization (i.e., boy–father, boy–mother, girl–father, and girl–mother CLPNs) to explore potential gender patterns in the transmission of depressive symptoms. The *glmnet* package (Friedman et al., 2010) was applied to estimate cross-lagged coefficients (from T1 to T2) between adolescents’ and parents’ depressive symptoms through a series of LASSO regressions. The optimal coefficients selected by the LASSO regression and 10-fold cross-validation methods resulted in a sparse and accurate network. The *qgraph* package was then employed to visualize the network results (Epskamp et al., 2012), where the nodes represent various depressive symptoms of adolescents and parents, the edges between the nodes of adolescents and their parents (i.e., bridging edge) indicate the depressive transmission, and the arrows on the edges denote the direction of transmission from T1 to T2.

Third, we calculated the centrality indices (IEI, OEI, and BEI) for each CLPN. In this study, IEI represents the extent to which a specific depressive symptom in adolescents or parents at T2 is influenced by all the other depressive symptoms at T1, while OEI reflects the impact of a specific depressive symptom at T1 on all the other depressive symptoms at T2. BEI indicates the influence of a specific depressive symptom at T1 in one group on all the depressive symptoms in the opposite group at T2. Specifically, if the node is from adolescents, it predicts all the depressive symptoms in parents; if the node is from parents, it predicts all the depressive symptoms in adolescents.

Fourth, we assessed the stability and accuracy of each CLPN and centrality index using the *bootnet* package (Epskamp & Fried, 2018). Network stability was evaluated by computing 95% confidence intervals (CIs) around each edge weight through nonparametric bootstrapping with 1000 iterations, and narrower CIs indicating greater reliability. The accuracy of the centrality index was evaluated by calculating the correlation stability (CS) coefficient, which measures the maximum proportion of cases that can be excluded while maintaining a correlation greater than 0.70 between the centrality index of the original network and that of the network estimated after excluding those cases. A CS coefficient above 0.25 is considered acceptable, while values exceeding 0.50 indicate high robustness.

Finally, we followed the guidelines for the intergroup comparison of CLPNs (Rhemtulla et al., 2017) to indicate the similarity or dissimilarity among the stable networks: (1) we estimated the correlation of the edge matrices and the proportion of edges with the same direction (positive, negative, and non) between networks; (2) we estimated the correlation of the centrality indices and compared the most central symptoms and strongest edges across networks.

## 3. Results

### 3.1. Comparing the Boy–Father CLPN and the Boy–Mother CLPN

**Figure 1:**
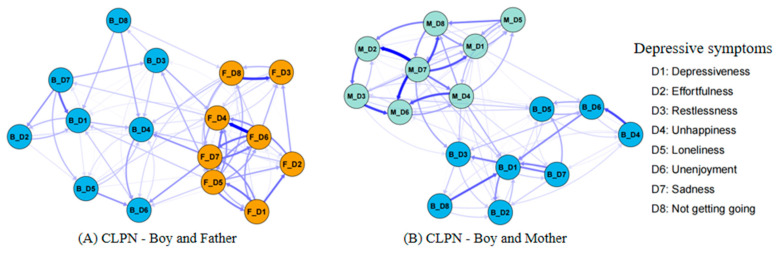
Cross-lagged panel network (CLPN) between boys and their parents. *Note.* The participants’ depressive symptoms were evaluated in eight items, including D1 depressiveness (“I felt depressed.”), D2 effortfulness (“I felt everything I did was an effort.”), D3 restlessness (“My sleep was restless.”), D4 unhappiness (“I was happy.”, reverse scored item), D5 loneliness (“I felt lonely.”), D6 unenjoyment (“I enjoyed life.”, reverse scored item), D7 sadness (“I felt sad.”), and D8 felt life could not go on (“I felt my life could not go on.”). Each item was self-reported on a 4-point scale, with scores ranging from 1 = *rarely or none of the time* to 4 = *most or all of the time*.

The CLPNs for boys and their fathers/mothers are presented in Figure 1. For network stability (Figure S1 in the Supplementary Material), the 95% bootstrapping confidence intervals (CIs) around the edge weights of these two CLPNs were small to moderate, indicating acceptable edge stability. For network accuracy (Figure S2 in the Supplementary Material), the CS coefficients of the OEI (boy–father = 0.28; boy–mother = 0.28) and the IEI (boy–father = 0.36; boy–mother = 0.28) were acceptable, whereas the CS coefficients of the BEI (boy–father = 0.13; boy–mother = 0.05) were unstable. The edge weight and centrality difference tests are shown in Figures S3 and S4 of the Supplementary Material, respectively. Therefore, we reported the findings on the edges, OEI, and IEI of these two CLPNs.

**Figure 2:**
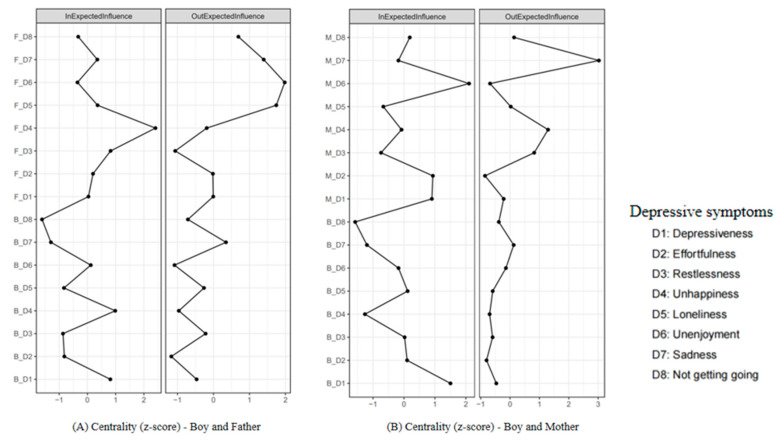
Standardized centrality indices of the cross-lagged panel network between boys and their parents. *Note.* The participants’ depressive symptoms were evaluated in eight items, including D1 depressiveness (“I felt depressed.”), D2 effortfulness (“I felt everything I did was an effort.”), D3 restlessness (“My sleep was restless.”), D4 unhappiness (“I was happy.”, reverse scored item), D5 loneliness (“I felt lonely.”), D6 unenjoyment (“I enjoyed life.”, reverse scored item), D7 sadness (“I felt sad.”), and D8 felt life could not go on (“I felt my life could not go on.”). Each item was self-reported on a 4-point scale, with scores ranging from 1 = *rarely or none of the time* to 4 = *most or all of the time*.

For the edges, the edge matrices of the boy–father CLPN (Figure 1A and Table S1) and the boy–mother CLPN (Figure 1B and Table S2) were not significantly correlated (*r* = −0.03, *p* = 0.736), with 41.8% of the edges having different directions. The strongest bridging edges of these two CLPNs were also different (Table 3), with the edge from *father felt sad* (F_D7) to *boy felt unhappy* (B_D4; *β* = 0.08) and the edge from *mother felt sad* (M_D7) to *boy slept restlessly* (B_D3; *β* = 0.06) showing the highest scores, respectively. For the central symptoms, the OEI of the boy–father CLPN (Figure 2A) and the boy–mother CLPN (Figure 2B) were also not significantly correlated (*r* = 0.28, *p* = 0.299). However, in these two CLPNs, the parents’ depressive symptoms had higher influences than those of the boys, with the *father did not enjoy life* (F_D6 = 1.98) and the *mother felt sad* (M_D7 = 3.03) having the highest OEI. Similarly, the IEI of these two CLPNs were not significantly correlated (*r* = −0.02, *p* = 0.951), with the *father felt unhappy* (F_D4 = 2.41) and the *mother did not enjoy life* (M_D6 = 2.10) exhibiting the highest scores, respectively.

### 3.2. Comparing the Girl–Father CLPN and the Girl–Mother CLPN

**Figure 3:**
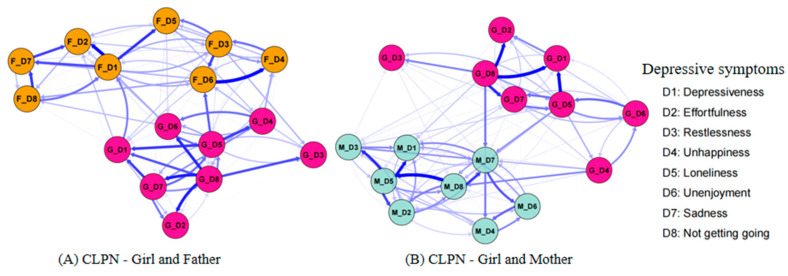
Cross-lagged panel network (CLPN) between girls and their parents. *Note.* The participants’ depressive symptoms were evaluated in eight items, including D1 depressiveness (“I felt depressed.”), D2 effortfulness (“I felt everything I did was an effort.”), D3 restlessness (“My sleep was restless.”), D4 unhappiness (“I was happy.”, reverse scored item), D5 loneliness (“I felt lonely.”), D6 unenjoyment (“I enjoyed life.”, reverse scored item), D7 sadness (“I felt sad.”), and D8 felt life could not go on (“I felt my life could not go on.”). Each item was self-reported on a 4-point scale, with scores ranging from 1 = *rarely or none of the time* to 4 = *most or all of the time*.

The CLPNs for girls and their fathers/mothers are presented in Figure 3. For network stability (Figure S5 in the Supplementary Material), the 95% bootstrapping confidence intervals (CIs) around the edge weights of these two CLPNs were small to moderate, indicating acceptable edge stability. For network accuracy (Figure S6 in the Supplementary Material), the CS coefficients of the OEI (girl–father = 0.28; girl–mother = 0.36) and the IEI (girl–father = 0.28; girl–mother = 0.28) were acceptable, whereas the CS coefficients of the BEI (girl–father = 0.05; girl–mother = 0.13) were unstable. The edge weight and centrality difference tests were shown in Figures S7 and S8 of the Supplementary Material, respectively. Therefore, we similarly reported the results on the edges, OEI, and IEI of these two CLPNs.

**Figure 4:**
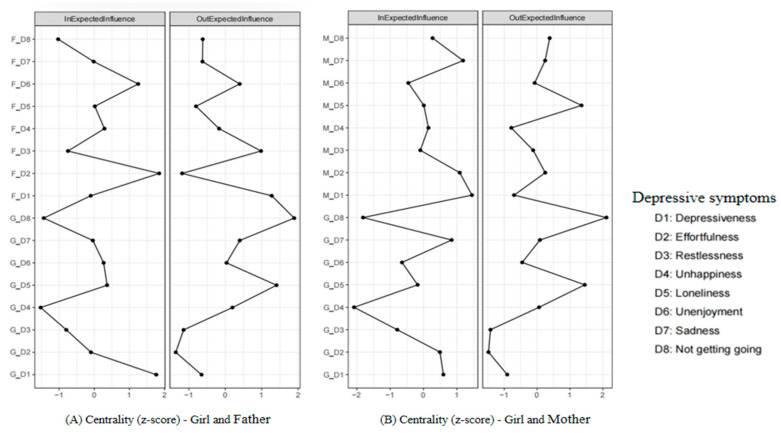
Standardized centrality indices of the cross-lagged panel network between girls and their parents. *Note.* The participants’ depressive symptoms were evaluated in eight items, including D1 depressiveness (“I felt depressed.”), D2 effortfulness (“I felt everything I did was an effort.”), D3 restlessness (“My sleep was restless.”), D4 unhappiness (“I was happy.”, reverse scored item), D5 loneliness (“I felt lonely.”), D6 unenjoyment (“I enjoyed life.”, reverse scored item), D7 sadness (“I felt sad.”), and D8 felt life could not go on (“I felt my life could not go on.”). Each item was self-reported on a 4-point scale, with scores ranging from 1 = *rarely or none of the time* to 4 = *most or all of the time*.

For the edges, the edge matrices between the girl–father CLPN (Figure 3A and Table S3) and the girl–mother CLPN (Figure 3B and Table S4) were not significantly correlated (*r* = −0.05, *p* = 0.604), with 37.7% of the edges having different directions. The strongest bridging edges of these two CLPNs were also different (see Table 4), with the edge from *girl felt lonely* (G_D5) to *father did not enjoy life* (F_D6; *β* = 0.10) and the edge from *girl felt life could not go on* (G_D8) to *mother felt sad* (M_D7; *β* = 0.09) showing the highest scores, respectively. For the central symptoms, the OEI of the girl–father CLPN (Figure 4A) and the girl–mother CLPN (Figure 4B) were significantly correlated (*r* = 0.51, *p* = 0.044). In these two CLPNs, the girl’s depressive symptoms had higher influences than the symptoms of the parents, with the *girl felt life could not go on* (G_D8 = 1.89) and *felt lonely* (G_D5 = 1.41) having the highest OEI. The IEI of these two CLPNs were also significantly correlated (*r* = 0.54, *p* = 0.027), but the highest IEI of them were different, with the *father felt everything was an effort* (F_D2 = 1.84) and the *mother felt depressed* (M_D1 = 1.45) exhibiting the highest scores, respectively.

### 3.3. Comparing the Boy–Father CLPN and the Girl–Father CLPN

The edge matrices between the boy–father CLPN (Figure 1A and Table S1) and the girl–father CLPN (Figure 3A and Table S3) were not significantly correlated (*r* = 0.01, *p* = 0.898), with 37.5% of the edges having different directions. Furthermore, the strongest bridging edges of the boy–father CLPN (see Table 3) and the girl–father CLPN (see Table 4) were different. Specifically, boys were more vulnerable to being influenced by their fathers, such as *boy felt unhappy* (B_D4) could be predicted by *father felt sad* (F_D7; *β* = 0.08). Conversely, girls had a greater influence on their fathers, such as *girl felt lonely* (G_D5) predicted *father did not enjoy life* (F_D6; *β* = 0.10).

The central symptoms of the boy–father CLPN (Figure 2A) and the girl–father CLPN (Figure 4A) were also not significantly correlated (OEI: *r* = −0.05, *p* = 0.848; IEI: *r* = 0.18, *p* = 0.483). The fathers’ depressive symptoms had a greater impact on the boy–father CLPN, with the *father did not enjoy life* (F_D6 = 1.98) showing the highest OEI. Conversely, the depressive symptoms of the girls had a higher influence on the girl–father CLPN, with the *girl felt life could not go on* (G_D8 = 1.89) showing the highest OEI. Furthermore, the IEI of these two CLPNs were different, with the *father felt unhappy* (F_D4 = 2.41) of the boy–father CLPN and the *father felt everything was an effort* (F_D2 = 1.84) of the girl–father CLPN having the highest scores, respectively.

### 3.4. Comparing the Boy–Mother CLPN and the Girl–Mother CLPN

The edge matrices between the boy–mother CLPN (Figure 1B and Table S2) and the girl–mother CLPN (Figure 3B and Table S4) were not significantly correlated (*r* = −0.01, *p* = 0.300), with 35.2% of the edges having different directions. Furthermore, the strongest bridging edges of the boy–mother CLPN (see Table 3) and the girl–mother CLPN (see Table 4) were different. Specifically, boys were more vulnerable to being influenced by their mothers, such as *boy slept restlessly* (B_D3) could be predicted by *mother felt sad* (M_D7; *β* = 0.06). Conversely, girls had a greater tendency to influence their mothers, such as *girl felt life could not go on* (G_D8) predicted *mother felt sad* (M_D7; *β* = 0.09).

The central symptoms of the boy–mother CLPN (Figure 2B) and the girl–mother CLPN (Figure 4B) were also not significantly correlated (OEI: *r* = −0.14, *p* = 0.601; IEI: *r* = 0.38, *p* = 0.138). The mothers’ depressive symptoms had a greater impact on the boy–mother CLPN, with the *mother felt sad* (M_D7 = 3.03) showing the highest OEI. Conversely, the depressive symptoms of the girls had a higher influence on the girl–mother CLPN, with the *girl felt life could not go on* (G_D8 = 2.11) showing the highest OEI. Furthermore, the IEI of these two CLPNs were different, with the *mother did not enjoy life* (M_D6 = 2.10) of the boy–mother CLPN and the *mother felt depressed* (M_D1 = 1.45) of the girl–mother CLPN showing the highest scores, respectively.

## 4. Discussion

The present study explored the gender-specific transmission of depressive symptoms in Chinese families. Consistent with our hypothesis, each CLPN, based on gender categorization, exhibited a relatively stable pattern of depressive symptoms between the adolescents and parents. Additionally, by comparing CLPNs, we identified both homogeneity and heterogeneity in the transmission of depression across gender patterns, with boys being more susceptible to parental influence, and girls more likely to influence their parents.

### 4.1. Comparing the Parental Influences on Boys and Girls

Regarding the CLPNs between boys and their parents, the *father did not enjoy life* (F_D6) and *felt lonely* (F_D5) as well as the *mother felt sad* (M_D7) and *felt unhappy* (M_D4) exhibited significant influences within the networks, respectively. Symptoms of “loneliness” and “unenjoyment” are often internalized emotional experiences, which are typically not easily observable by others (Cacioppo & Patrick, 2008). In contrast, “sadness” and “unhappiness” are more frequently accompanied by external expressions, making them more observable (Gross, 2002). In Chinese culture, fathers are typically seen as stern and reserved (Liu & Yu, 2024), while mothers are often perceived as nurturing and emotionally supportive (Kan & He, 2018). This difference in role characteristics may explain why fathers tend to transmit more implicit symptoms, in contrast to mothers, who tend to transmit more explicit symptoms in the network. Notably, our results of network edges also indicated that the *father felt sad* (F_D7) could predict *boy felt unhappy* (B_D4) and *did not enjoy life* (B_D6). Given the complexity of emotional expression (Wang et al., 2025; Werner-Seidler et al., 2020), this phenomenon may suggest that paternal “sadness” could be hidden in internal symptoms or masked by outwardly optimistic appearances, indirectly affecting children.

Additionally, several bridging edges (e.g., F_D5/M_D7 → B_D3) revealed that boys’ sleep quality is particularly vulnerable to being influenced by parental “sadness” and “unhappiness”, which aligns with previous studies (de Jong et al., 2016; El-Sheikh et al., 2012). This finding re-emphasizes the impact of family environment on adolescents’ sleep (El-Sheikh & Kelly, 2017). The bridging edge from *boy slept restlessly* (B_D3) to *father felt unhappy* (F_D4) suggested that ”restlessness” in boys could, in turn, affect their fathers, which may reflect depressive symptom outcomes related to paternal concerns for their children (Sifaki et al., 2021). Therefore, poor sleep in boys may serve as the mediated symptom in the family symptoms network, and improvements in sleep quality may help prevent the subsequent physical and mental problems not only in children themselves (Chen et al., 2025; Liang et al., 2023) but also in their family members.

In terms of the CLPNs between girls and their parents, the *girl felt life could not go on* (G_D8) and *felt lonely* (G_D5) had the highest impact in both networks. During adolescence, girls typically face many challenges, such as body image concerns mediated by physiological factors (Choukas-Bradley et al., 2022) and heightened sensitivity to peer relationships and social evaluation (Potter & Yoon, 2023). These challenges may increase girls’ cognitive and emotional loads, further contributing to their “felt life could not go on” and “unhappiness”. Furthermore, a series of bridging edges indicated that the symptoms of girls could strongly predict their paternal “unenjoyment” and maternal “sadness”, which is not identified by X. Gong et al. (2024). This discrepancy may be due to the fact that their study was based on early adolescents (*M*_age_ = 11.30 years), whereas the present study focused on a wider range of girls (*M*_age_ = 13.85 years, range 9–16 years), which is probably more generalizable. Therefore, the physical and mental development of daughters remains closely linked to the depressive symptoms of their parents, and the direct mediation on paternal “depressiveness” should be taken seriously.

It is worth noting that “loneliness” emerges as a core symptom in these networks. Gender role expectations (e.g., female obedience and propriety; male independence and strength; Langer et al., 2019; Leaper & Friedman, 2007), particularly in China, may encourage parents to adopt harsh criticism and control to ensure their children meet societal standards (Luo et al., 2013). Such over-concern could elicit confrontation between adolescents and their parents, reducing effective communication and thereby resulting in mutual “loneliness” (Kakihara et al., 2010). The “loneliness” not only triggers “sleep restlessness” and “unhappiness” in the current networks, but also causes other symptoms, including self-injury (Guo et al., 2022) and addictive behaviors (Moretta et al., 2023). To intervene in loneliness, reactivating honest and empathetic communication may be an effective and affordable way (Lloyd et al., 2023), which requires the joint efforts of family members and the support of mental health agencies, schools, and communities.

### 4.2. Comparing the Influences of Boys and Girls on Parents

Regarding the CLPNs between boys/girls and their parents, parental symptoms had a greater impact on boys, while the symptoms of girls had a greater impact on parents. One possible explanation is that girls are more emotionally sensitive and contagious than boys (Chaplin, 2015; Demkowicz et al., 2025), which may be due to a series of factors. For example, the emotional interactions of boys and girls with their parents are inconsistent, with girls more likely to rely on parental support for emotional regulation, while boys prefer to cope independently (Do et al., 2025; Rose & Rudolph, 2006). In addition, girls tend to mature earlier (Brix et al., 2019; Huang et al., 2025), which may make their depressive symptoms more influenced by factors outside the home, such as body image concerns (Zimmer-Gembeck et al., 2018) and academic stress (Vist Hagen et al., 2022). It is also interesting that the same symptoms are transmitted between fathers and girls (e.g., G_D1 → F_D1 and F_D3 → G_D3), which are not observed in boys. This phenomenon may be attributed to the unique nature of the father–daughter relationship, which tends to have a deeper emotional connection compared to the father–son relationship (Dmitrieva & Espel, 2023).

Another possible explanation is that parents have higher expectations for their sons than daughters in some Chinese families with strong traditional values (Liu et al., 2021). There is a well-known saying in Chinese culture, “Hope the son will be a loong”, which implies that parents strive for their sons to become capable and accomplished through various efforts. However, this also means that parents are at high risk of transmitting negative symptoms and perceptions to their sons during the interaction, as indicated by a series of bridging edges linking boys to their parents. In particular, maternal “sadness” and “unhappiness” are predictive of boys’ “sleep restlessness” and “depressiveness”, which may reflect boys’ responsibility and anxiety for their mother’s negative state (Hwang, 1987; Klimes-Dougan & Bolger, 1998), further mediating their poorer sleep quality and depression. Considering that both mothers’ and daughters’ symptoms are highly influential in the family symptom network, increased attention on the mental health of females in the family and the development of related social and biological interventions will help to cut off the transmission of depression at its source.

### 4.3. Irregular IEI and Unstable BEI

The IEI of these CLPNs showed that both the parents’ and adolescents’ symptoms were affected in the familial network (e.g., F_D4 and B_D4 have high IEI on the boy–father CLPN), rather than one side being more strongly influenced. The unstable BEI also supported that a range of symptoms are not simply and directly influenced from one side to the other, and that transmission may be mediated by a series of factors. For example, parental depression may mediate overcontrol or emotional neglect leading to adolescents’ externalizing problems, which, in turn, exacerbate family conflicts and aggravate parental depression (Korhonen et al., 2014; Luo et al., 2023). Additionally, depressive symptoms may be transmitted by affecting brain structures (Butterfield et al., 2021; Cattarinussi et al., 2021; W. Gong et al., 2021), but the specific pattern of biological interactions between parents and adolescents remains largely unclear. Given that the present study identified certain gender differences in the transmission of depression, future studies could incorporate flow networks (Epskamp et al., 2012; Fan et al., 2024) to further explore the mediating biological and social mechanisms.

### 4.4. Theoretical and Practical Implications

In general, our findings on gender-specific CLPNs provide several theoretical and practical implications. For theoretical implications, this study contributed to the intergenerational transmission (Branje et al., 2020) and family systems theories (Cox & Paley, 2003). The edges between depressive symptoms in parents and children reaffirm the reciprocity of transmission, highlighting the complexity of the family system and the need to explore the mechanisms involved. Gender emerges as a significant factor influencing symptom-level transmission, underscoring the value of gender-specific interventions to mitigate the aggravation of depression within families. For practical implications, our study provides detailed insights into the interventions for depressive symptoms, especially for Chinese families. We should increase concern for female mental health in the family (e.g., timely alleviation of depressive symptoms in mothers or daughters), which can help cut off the transmission of depressive symptoms earlier. Furthermore, honest and empathetic communication between parents and children should be widely encouraged, thereby effectively reducing “loneliness” and the range of symptoms it mediates.

### 4.5. Limitations and Future Directions

This study has a number of limitations. First, to maintain a sufficient sample, the present analysis included only two time points of data in the CFPS, which may incompletely represent the transmission of depressive symptoms over time. Future studies should incorporate more data across different stages to further explore the cycle of depressive symptoms within families. Second, the present study focused only on eight depressive symptoms, neglecting other important symptoms, such as suicide (Hamilton, 1960), cognitive symptoms (Teng et al., 2023), addiction (Teng et al., 2024), and externalizing problems (X. Zhang et al., 2025). Moreover, this exploration was based on a nationally representative sample from China, representing a Chinese cultural perspective. Given that different cultures may have different emotional expressions (Soto et al., 2005), it is necessary to consider more depressive symptoms from diverse backgrounds to further test the familial depressive transmission networks and their associated mechanisms.

## 5. Conclusions

Building on data from the CFPS and analysis of the CLPNs, the present study identified certain homogeneity and heterogeneity in the transmission of depressive symptoms based on gender categorization. Notably, “loneliness” repeatedly showed the strongest impacts on other symptoms across familial networks. Furthermore, the symptoms of girls are more likely to influence their parents, while the symptoms of boys, especially the “sleep restlessness”, were susceptible to parental influence. Therefore, increased concerns for female mental health, communication, and improvement of sleep may help to effectively cut off the transmission of depression and enhance psychological well-being.

## Figures and Tables

**Table 1:** Descriptive statistics for demographic characteristics.

Characteristics	Adolescent Boys				Adolescent Girls			
	*M* (*SD*)		*N*		*M* (*SD*)		*N*	
Age, years, T1	13.76 (2.58)		760		13.85 (2.56)		709	
Age, years, T2	15.64 (2.54)		760		15.59 (2.57)		709	
Annual Family income, RMB, T1	104,819 (128,462)		706		103,403 (119,999)		700	
Annual Family income, RMB, T2	116,172 (129,738)		699		119,648 (203,038)		639	
Ethnicity	*n* (%)				*n* (%)			
Han	665 (87.50%)				614 (86.60%)			
non-Han	95 (12.50%)				95 (13.40%)			
	Maternal level		Paternal level		Maternal level		Paternal level	
	*M* (*SD*)	*N*	*M* (*SD*)	*N*	*M* (*SD*)	*N*	*M* (*SD*)	*N*
Age, years, T1	38.12 (5.34)	760	41.36 (5.58)	688	37.87 (5.15)	709	40.87 (5.42)	612
Age, years, T2	39.98 (5.38)	760	43.11 (5.57)	688	39.74 (5.12)	760	42.76 (5.39)	612
Educational level	*n* (%)				*n* (%)			
Primary school and below	323 (42.50%)		297 (43.17%)		276 (38.93%)		262 (38.08%)	
Secondary school	207 (27.23%)		188 (27.33%)		200 (28.21%)		175 (25.44%)	
Post-secondary	100 (13.16%)		103 (14.97%)		113 (15.94%)		96 (13.95%)	
Bachelor and above	70 (9.21%)		51 (7.41%)		44 (6.21%)		24 (3.49%)	

*Note.* Given that the participants’ ethnicity, maternal educational level, and paternal educational level are consistent across years, the table only presents these variables collected at T1.

**Table 2:** Descriptive statistics for depressive symptoms.

Depressive Symptoms	Boy–Parent Cross-Lagged Panel Network						Girl–Parent Cross-Lagged Panel Network					
	Boy’s Levels *M* (*SD*)		Maternal Levels *M* (*SD*)		Paternal Levels *M* (*SD*)		Girl’s Levels *M* (*SD*)		Maternal Levels *M* (*SD*)		Paternal Levels *M* (*SD*)	
	T1	T2	T1	T2	T1	T2	T1	T2	T1	T2	T1	T2
D1	1.63 (0.75)	1.52 (0.70)	1.82 (0.76)	1.87 (0.75)	1.72 (0.76)	1.77 (0.78)	1.68 (0.76)	1.65 (0.75)	1.85 (0.82)	1.92 (0.82)	1.71 (0.77)	1.76 (0.79)
D2	1.54 (0.72)	1.45 (0.65)	1.71 (0.76)	1.75 (0.77)	1.68 (0.80)	1.71 (0.79)	1.56 (0.77)	1.60 (0.71)	1.73 (0.81)	1.78 (0.81)	1.69 (0.80)	1.78 (0.85)
D3	1.48 (0.76)	1.52 (0.80)	1.75 (0.87)	1.87 (0.92)	1.72 (0.89)	1.77 (0.88)	1.47 (0.78)	1.58 (0.80)	1.83 (0.92)	1.92 (0.92)	1.72 (0.86)	1.76 (0.90)
D4	1.85 (0.83)	1.87 (0.80)	2.10 (0.92)	2.04 (0.88)	2.09 (0.93)	2.07 (0.91)	1.78 (0.83)	1.79 (0.79)	2.08 (0.94)	2.09 (0.92)	2.01 (0.92)	2.06 (0.90)
D5	1.40 (0.70)	1.36 (0.63)	1.45 (0.65)	1.53 (0.71)	1.48 (0.72)	1.50 (0.72)	1.39 (0.67)	1.42 (0.66)	1.48 (0.72)	1.59 (0.79)	1.48 (0.74)	1.60 (0.81)
D6	1.73 (0.75)	1.75 (0.74)	2.00 (0.87)	1.88 (0.87)	1.97 (0.91)	1.93 (0.89)	1.65 (0.76)	1.69 (0.74)	1.98 (0.88)	1.98 (0.90)	1.92 (0.91)	1.96 (0.87)
D7	1.50 (0.69)	1.44 (0.65)	1.60 (0.64)	1.66 (0.68)	1.49 (0.67)	1.52 (0.63)	1.52 (0.68)	1.56 (0.69)	1.62 (0.69)	1.70 (0.77)	1.50 (0.66)	1.56 (0.74)
D8	1.12 (0.44)	1.12 (0.42)	1.24 (0.55)	1.29 (0.61)	1.18 (0.52)	1.22 (0.54)	1.15 (0.46)	1.18 (0.52)	1.25 (0.56)	1.32 (0.67)	1.22 (0.58)	1.29 (0.65)

*Note.* The participants’ depressive symptoms were evaluated in eight items, including D1 depressiveness (“I felt depressed.”), D2 effortfulness (“I felt everything I did was an effort.”), D3 restlessness (“My sleep was restless.”), D4 unhappiness (“I was happy.”, reverse scored item), D5 loneliness (“I felt lonely.”), D6 unenjoyment (“I enjoyed life.”, reverse scored item), D7 sadness (“I felt sad.”), and D8 felt life could not go on (“I felt my life could not go on.”). Each item was self-reported on a 4-point scale, with scores ranging from 1 = *rarely or none of the time* to 4 = *most or all of the time*. The Cronbach’s *α* for all the items was 0.76 in T1 and 0.77 in T2.

**Table 3:** Bridging edges wights of the boy–father and the boy–mother cross-lagged panel network (CLPN).

Boy–Father CLPN		Boy–Mother CLPN	
Measure	Edge Weight	Measure	Edge Weight
F_D7 → B_D4	0.08	M_D7 → B_D3	0.06
F_D7 → B_D6	0.08	M_D4 → B_D1	0.06
F_D5 → B_D3	0.06	M_D4 → B_D3	0.04
B_D5 → F_D3	0.06	M_D7 → B_D1	0.04
B_D3 → F_D4	0.06	M_D3 → B_D5	0.04
F_D2 → B_D4	0.05	B_D5 → M_D6	0.03
F_D8 → B_D4	0.05	M_D8 → B_D6	0.03
F_D5 → B_D1	0.05	M_D4 → B_D7	0.03
F_D4 → B_D7	0.04	M_D4 → B_D5	0.03
F_D5 → B_D6	0.04	M_D4 → B_D2	0.03
B_D8 → F_D8	0.04	M_D7 → B_D2	0.03
F_D8 → B_D6	0.04	M_D3 → B_D3	0.02

*Note.* The participants’ depressive symptoms were evaluated in eight items, including D1 depressiveness (“I felt depressed.”), D2 effortfulness (“I felt everything I did was an effort.”), D3 restlessness (“My sleep was restless.”), D4 unhappiness (“I was happy.”, reverse scored item), D5 loneliness (“I felt lonely.”), D6 unenjoyment (“I enjoyed life.”, reverse scored item), D7 sadness (“I felt sad.”), and D8 felt life could not go on (“I felt my life could not go on.”). Each item was self-reported on a 4-point scale, with scores ranging from 1 = *rarely or none of the time* to 4 = *most or all of the time.*

**Table 4:** Bridging edges wights of the girl–father and the girl–mother cross-lagged panel network (CLPN).

Girl–Father CLPN		Girl–Mother CLPN	
Measure	Edge Weight	Measure	Edge Weight
G_D5 → F_D6	0.10	G_D8 → M_D7	0.09
G_D1 → F_D1	0.08	G_D4 → M_D8	0.08
G_D4 → F_D4	0.06	G_D5 → M_D7	0.07
G_D6 → F_D1	0.05	G_D4 → M_D7	0.05
F_D3 → G_D3	0.05	G_D5 → M_D8	0.05
G_D4 → F_D6	0.05	G_D8 → M_D1	0.04
G_D6 → F_D8	0.04	G_D8 → M_D4	0.03
F_D5 → G_D4	0.04	M_D5 → G_D7	0.03
G_D1 → F_D2	0.04	G_D5 → M_D3	0.03
G_D6 → F_D2	0.04	G_D7 → M_D1	0.03
G_D5 → F_D4	0.04	G_D8 → M_D3	0.03
G_D3 → F_D6	0.03	G_D4 → M_D2	0.02

*Note.* The participants’ depressive symptoms were evaluated in eight items, including D1 depressiveness (“I felt depressed.”), D2 effortfulness (“I felt everything I did was an effort.”), D3 restlessness (“My sleep was restless.”), D4 unhappiness (“I was happy.”, reverse scored item), D5 loneliness (“I felt lonely.”), D6 unenjoyment (“I enjoyed life.”, reverse scored item), D7 sadness (“I felt sad.”), and D8 felt life could not go on (“I felt my life could not go on.”). Each item was self-reported on a 4-point scale, with scores ranging from 1 = *rarely or none of the time* to 4 = *most or all of the time.*

## Data Availability

The data for this study originate from China Family Panel Studies (CFPS), available in both Chinese and English. CFPS provides comprehensive user guidelines and resources. Access to the CFPS data requires an application, as direct sharing or redistribution by individuals is not permitted. Detailed application information and video instructions are available upon account registration at http://www.isss.pku.edu.cn/cfps/download/login, accessed on 15 November 2024.

## Supplementary Materials

The following supporting information can be downloaded at https://www.mdpi.com/article/10.3390/bs15050672/s1. Figure S1: Bootstrapped 95% confidence intervals around each edge weight of cross-lagged panel networks between boys and their parents; Figure S2: Stability of centrality measures for cross-lagged panel networks between boys and their parents; Figure S3: Edge weight difference tests for cross-lagged panel networks between boys and their parents; Figure S4: Centrality difference tests for cross-lagged panel networks between boys and their parents; Figure S5: Bootstrapped 95% confidence intervals around each edge weight of cross-lagged panel networks between girls and their parents; Figure S6: Stability of centrality measures for cross-lagged panel networks between girls and their parents; Figure S7: Edge weight difference tests for cross-lagged panel networks between girls and their parents; Figure S8: Centrality difference tests for cross-lagged panel networks between girls and their parents; Table S1: Edges matrix of the boy–father cross-lagged panel network; Table S2: Edges matrix of the boy–mother cross-lagged panel network; Table S3: Edges matrix of the girl–father cross-lagged panel network; Table S4: Edges matrix of the girl–mother cross-lagged panel network.

## Abbreviations

The following abbreviations are used in this manuscript:

CLPN	cross-lagged panel network
CFPS	China Family Panel Studies
IEI	in-expected influence
OEI	out-expected influence
BEI	bridge expected influence
CIs	confidence intervals
