# Supplementary material for: Gender-Specific Transmission of Depressive Symptoms in Chinese Families: A Cross-Lagged Panel Network Analysis Based on the China Family Panel Studies
Source: Behav Sci (Basel). 2025 May 14;15(5):672. doi: 10.3390/bs15050672 (PMC12109378; doi:10.3390/bs15050672)
Supplement: Supplementary file 1 [file behavsci-15-00672-s001.zip › behavsci-3521658-supplementary.pdf]

Supplementary materials

Gender-specific transmission of depressive symptoms in Chinese families: A cross-lagged panel network analysis based on the China Family Panel Studies

Cross-lagged panel networks between boys and their parents:

Figure S1. Bootstrapped 95% confidence intervals around each edge weight.

Figure S2. Stability of centrality measures.

Figure S3. Edge weight difference tests.

Figure S4. Centrality difference tests.

Table S1. Edges matrix of the boy-father cross-lagged panel network.

Table S2. Edges matrix of the boy-mother cross-lagged panel network.

Cross-lagged panel networks between girls and their parents:

Figure S5. Bootstrapped 95% confidence intervals around each edge weight.

Figure S6. Stability of centrality measures.

Figure S7. Edge weight difference tests.

Figure S8. Centrality difference tests.

Table S3. Edges matrix of the girl-father cross-lagged panel network.

Table S4. Edges matrix of the girl-mother cross-lagged panel network.

Figure S1. Bootstrapped 95% confidence intervals around each edge weight of cross-lagged panel networks between boys and their parents.

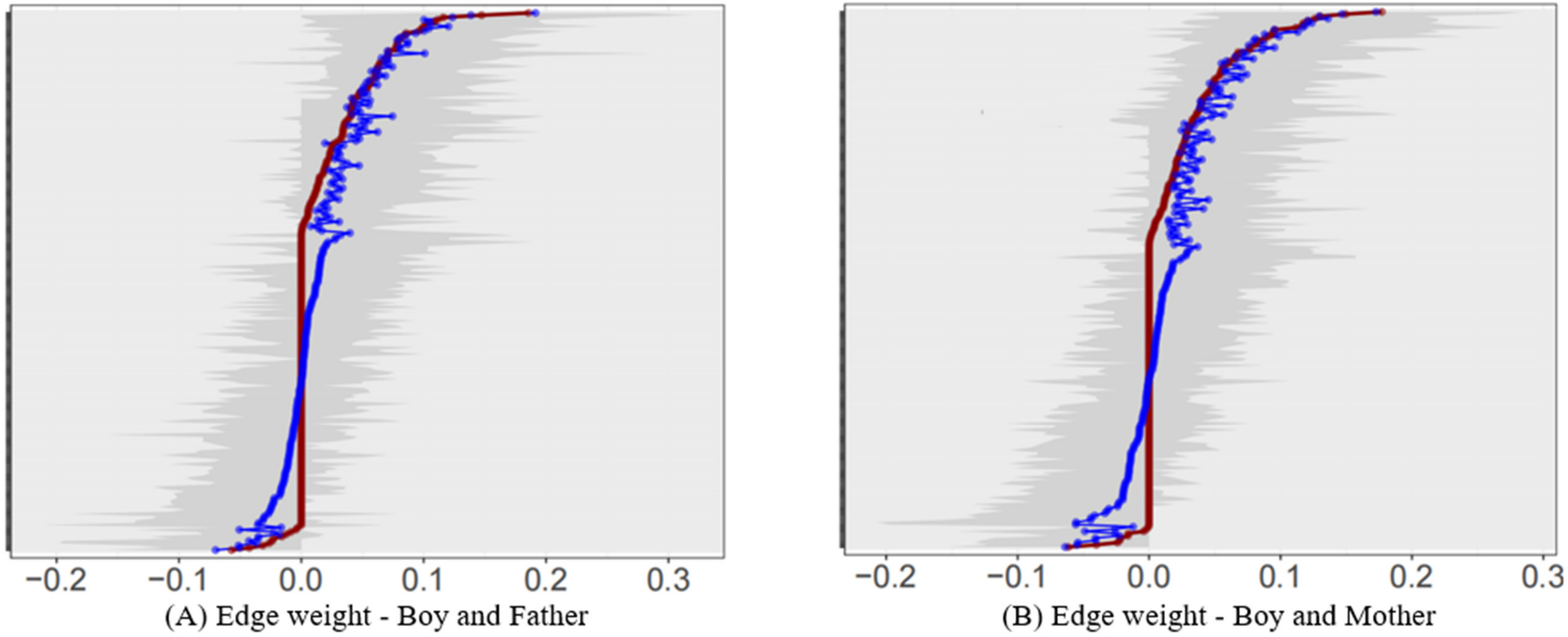

Note. The red line shows the edge weight in the estimated sample network and the gray area indicates the 95% bootstrapped confidence intervals.

Figure S2. Stability of centrality measures for cross-lagged panel networks between boys and their parents.

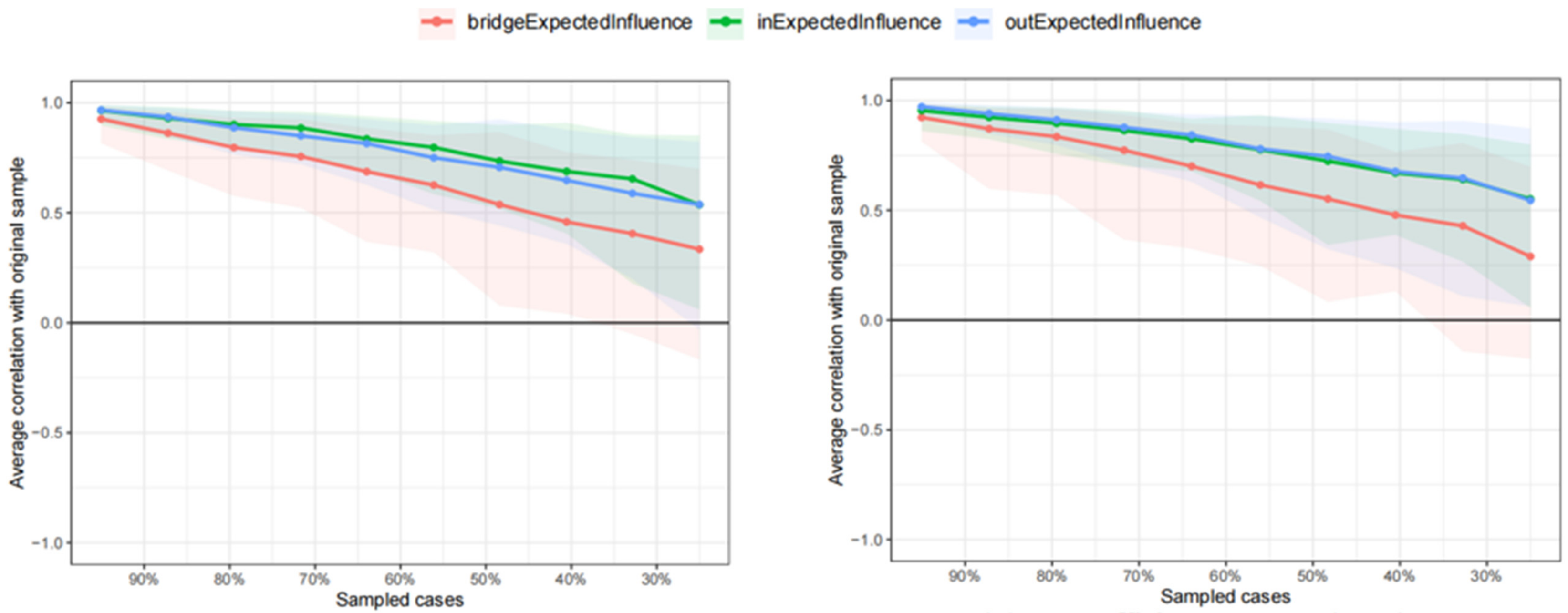

(A) CS coefficients - Boy and Father

(B) CS coefficients - Boy and Mother

Figure S3. Edge weight difference tests for cross-lagged panel networks between boys and their parents.

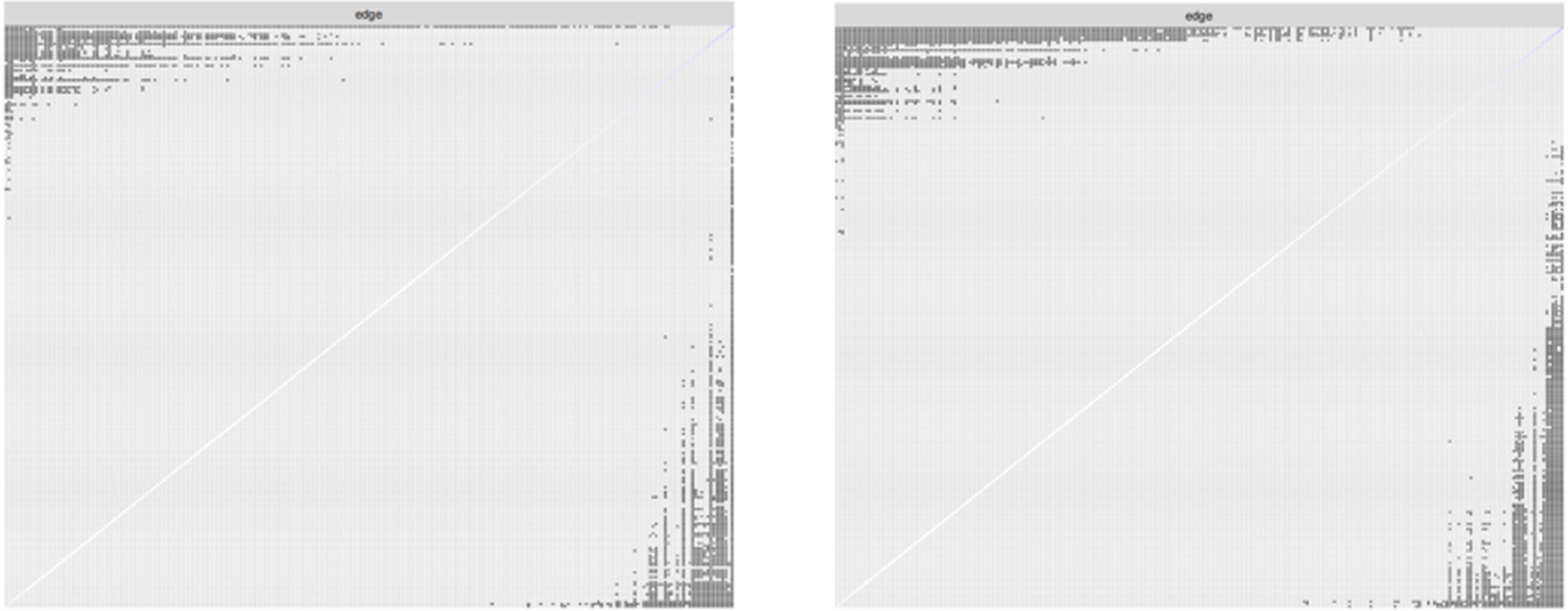

(A) Edge weight difference - Boy and Father (B) Edge weight difference - Boy and Mother

Note. Black boxes indicate edges that significantly differ from each other ($p < .05$), and gray boxes indicate edges that do not significantly differ.

Figure S4. Centrality difference tests for cross-lagged panel networks between boys and their parents.

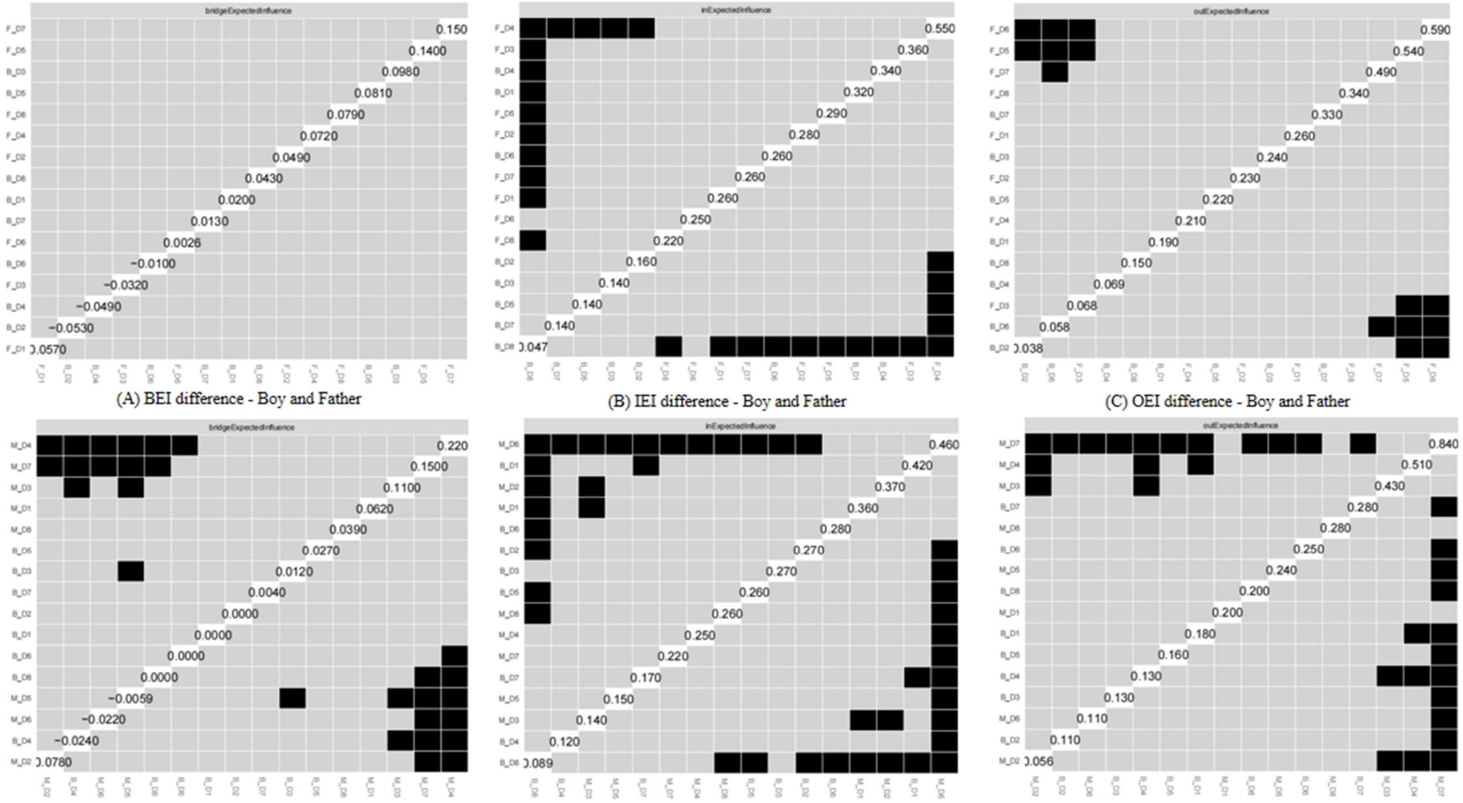

(A) BEI difference - Boy and Father (B) IEI difference - Boy and Father (C) OEI difference - Boy and Father

(D) BEI difference - Boy and Mother (E) IEI difference - Boy and Mother (F) OEI difference - Boy and Mother

Note. Black boxes indicate symptoms that significantly differ in centrality ($p < .05$), and gray boxes indicate symptoms whose centrality does not significantly differ.

Figure S5. Bootstrapped 95% confidence intervals around each edge weight of cross-lagged panel networks between girls and their parents.

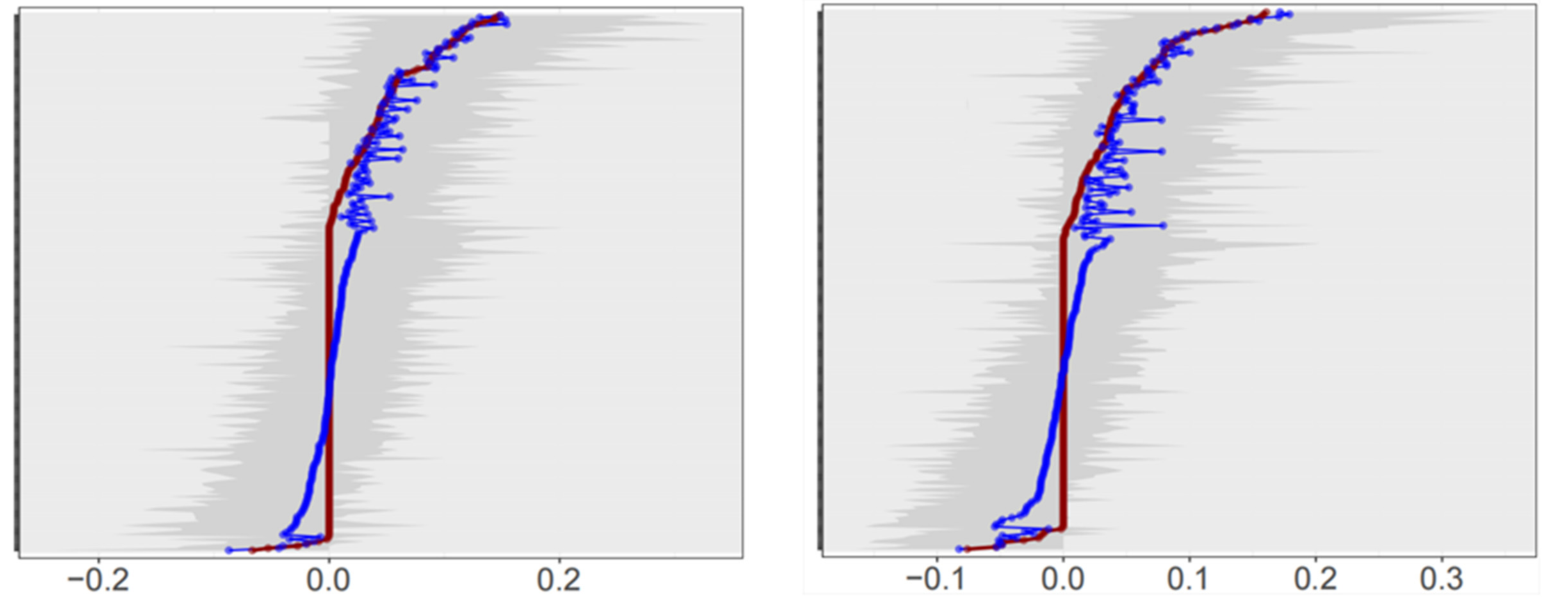

(A) Edge weight - Girl and Father (B) Edge weight - Girl and Mother

Note. The red line shows the edge weight in the estimated sample network and the gray area indicates the 95% bootstrapped confidence intervals.

Figure S6. Stability of centrality measures for cross-lagged panel networks between girls and their parents.

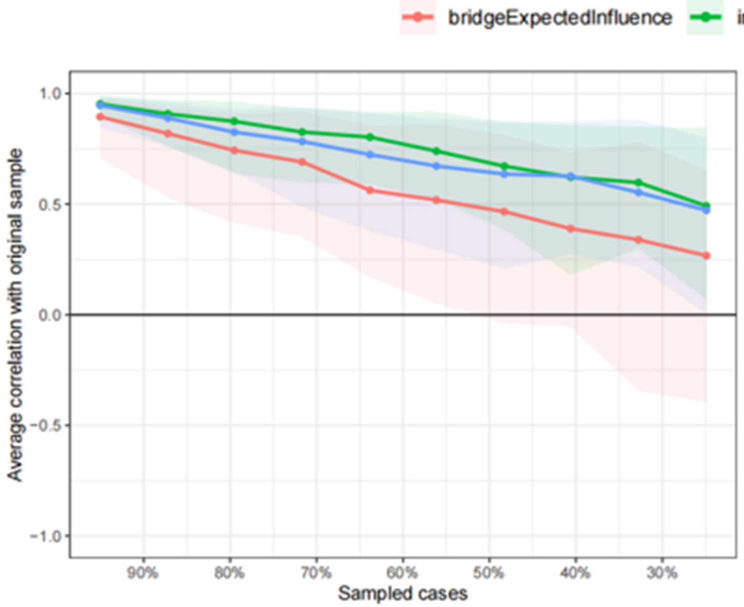

(A) CS coefficients - Girl and Father

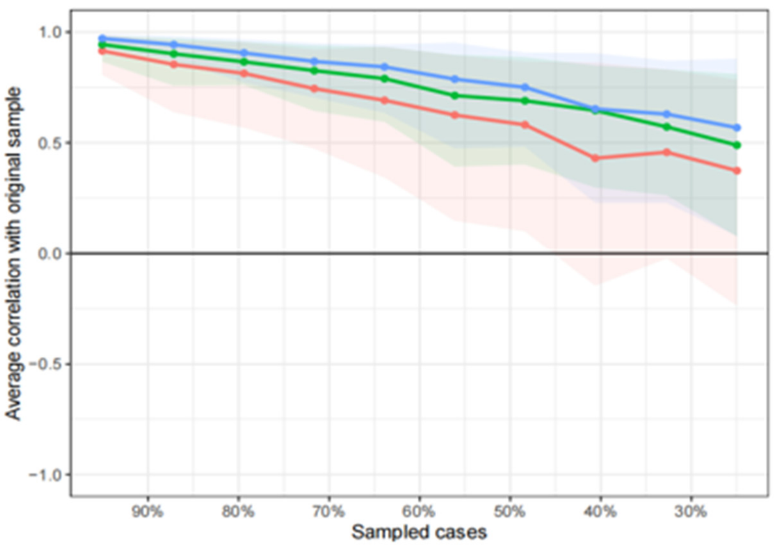

(B) CS coefficients - Girl and Mother

Figure S7. Edge weight difference tests for cross-lagged panel networks between girls and their parents.

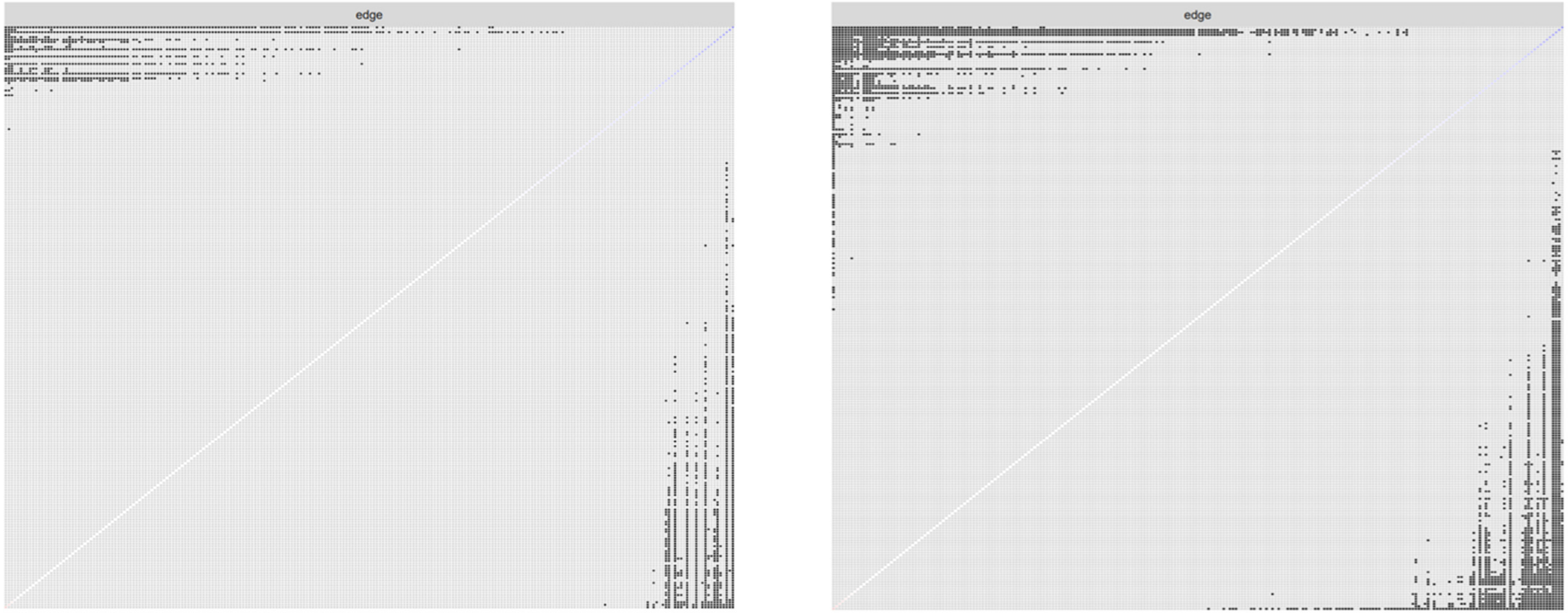

(A) Edge weight difference - Girl and Father (B) Edge weight difference - Girl and Mother

Note. Black boxes indicate edges that significantly differ from each other ($p < .05$), and gray boxes indicate edges that do not significantly differ.

Figure S8. Centrality difference tests for cross-lagged panel networks between girls and their parents.

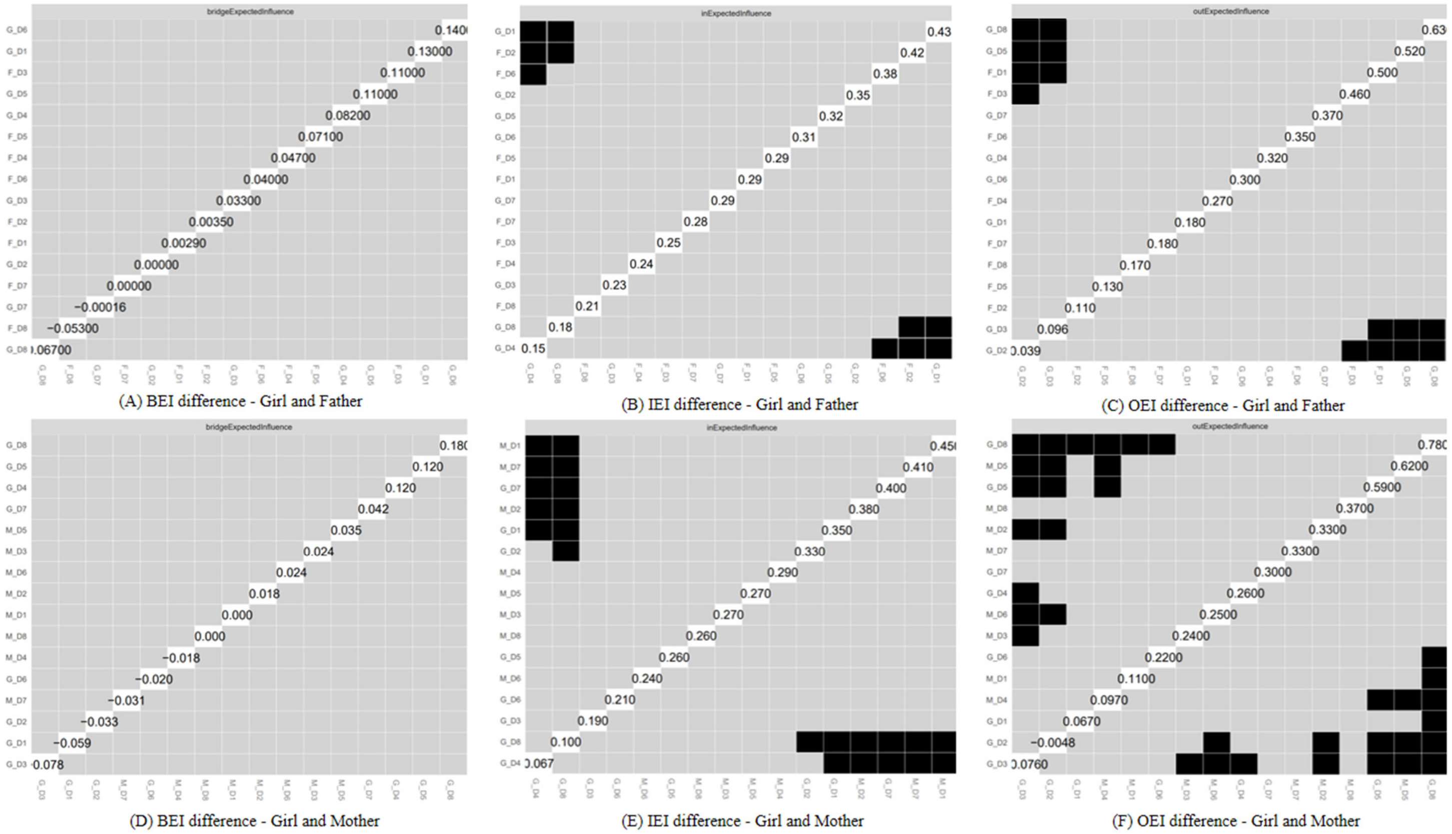

(A) BEI difference - Girl and Father

(B) IEI difference - Girl and Father

(C) OEI difference - Girl and Father

(D) BEI difference - Girl and Mother

(E) IEI difference - Girl and Mother

(F) OEI difference - Girl and Mother

Note. Black boxes indicate symptoms that significantly differ in centrality ($p < .05$), and gray boxes indicate symptoms whose centrality does not significantly differ.

Table S1. Edges matrix of the boy-father cross-lagged panel network.

	B_D1	B_D2	B_D3	B_D4	B_D5	B_D6	B_D7	B_D8	F_D1	F_D2	F_D3	F_D4	F_D5	F_D6	F_D7	F_D8
**B_D1**	0.02	0.05	0.00	0.05	0.02	0.00	0.02	0.00	0.00	0.00	0.00	0.02	0.00	0.00	-0.01	0.00
**B_D2**	0.06	0.02	0.00	0.01	0.02	0.00	0.00	0.00	0.00	0.00	0.00	0.03	-0.04	0.00	-0.01	-0.03
**B_D3**	0.01	0.01	0.10	0.05	0.01	0.02	0.00	0.04	0.00	0.00	0.00	0.06	0.00	0.01	0.00	0.00
**B_D4**	0.00	0.00	0.00	0.05	0.03	0.07	0.01	0.00	0.00	0.00	0.00	-0.03	0.00	0.00	0.00	0.00
**B_D5**	0.00	0.00	0.00	0.04	0.03	0.08	0.01	0.00	0.00	0.02	0.06	0.00	0.00	0.00	0.00	0.02
**B_D6**	0.04	0.00	0.00	0.03	0.00	0.06	0.00	0.00	0.00	0.00	0.00	-0.02	0.00	0.00	0.00	0.00
**B_D7**	0.11	0.08	0.06	0.00	0.05	0.00	0.11	0.00	0.00	0.00	0.00	0.00	0.00	0.00	0.00	0.00
**B_D8**	0.05	0.00	0.00	0.06	0.00	0.00	0.00	0.00	0.00	0.00	0.00	0.00	0.00	0.00	-0.03	0.04
**F_D1**	0.00	0.00	0.00	-0.01	0.00	-0.06	0.00	0.00	0.21	0.11	0.00	0.00	0.10	0.02	0.08	0.00
**F_D2**	0.01	0.00	0.00	0.05	0.00	0.01	0.00	0.00	0.00	0.14	0.07	0.09	0.00	0.00	0.02	0.00
**F_D3**	0.00	0.00	0.00	-0.04	0.00	-0.01	0.00	0.00	0.00	0.00	0.31	0.03	0.00	0.00	0.02	0.06
**F_D4**	0.02	0.00	0.00	0.00	0.00	0.00	0.04	0.00	0.00	0.02	0.03	0.08	0.07	0.00	0.03	0.00
**F_D5**	0.05	0.00	0.06	0.00	0.00	0.04	0.00	0.00	0.09	0.07	0.00	0.08	0.14	0.07	0.03	0.06
**F_D6**	0.00	0.00	0.01	0.00	0.00	-0.01	0.00	0.00	0.07	0.04	0.06	0.19	0.08	0.16	0.11	0.04
**F_D7**	0.00	0.00	0.00	0.08	0.00	0.08	0.00	0.00	0.06	0.00	0.01	0.10	0.03	0.10	0.18	0.02
**F_D8**	0.00	0.00	0.00	0.05	0.00	0.04	0.00	0.00	0.03	0.01	0.14	0.00	0.04	0.00	0.06	0.18

Note. Independent variables (i.e., predictors) are in columns, and dependent variables are in rows. Participants' depressive symptoms were evaluated in eight items, including D1 depressiveness ("I felt depressed."), D2 effortfulness ("I felt everything I did was an effort."), D3 restlessness ("My sleep was restless."), D4 unhappiness ("I was happy.", reverse scored item), D5 loneliness ("I felt lonely."), D6 unenjoyment ("I enjoyed life.", reverse scored item), D7 sadness ("I felt sad."), and D8 felt life could not go on ("I felt my life could not go on."). Each item was self-reported on a 4-point scale, with score ranging from 1 = *rarely or none of the time* to 4 = *most or all of the time*.

Table S2. Edges matrix of the boy-mother cross-lagged panel network.

	B_D1	B_D2	B_D3	B_D4	B_D5	B_D6	B_D7	B_D8	M_D1	M_D2	M_D3	M_D4	M_D5	M_D6	M_D7	M_D8
**B_D1**	0.07	0.08	0.00	0.01	0.03	0.00	0.04	0.00	0.00	0.00	0.00	0.00	0.00	0.00	0.00	0.00
**B_D2**	0.05	0.04	0.02	0.00	0.03	0.00	0.00	0.00	0.00	0.00	0.00	0.00	0.00	0.00	0.00	0.00
**B_D3**	0.01	0.04	0.09	0.00	0.02	0.00	0.00	0.04	0.00	0.00	0.01	0.00	0.00	0.01	0.01	0.00
**B_D4**	0.01	0.00	-0.05	0.10	0.02	0.12	0.02	0.02	-0.02	0.00	0.00	0.00	0.00	0.00	0.00	-0.01
**B_D5**	0.00	0.00	0.00	0.03	0.03	0.06	0.02	0.00	0.00	0.00	0.00	0.00	0.00	0.03	0.00	-0.01
**B_D6**	0.08	0.00	0.05	0.03	0.06	0.06	0.00	0.00	0.00	0.00	0.00	0.00	0.00	0.00	0.00	0.00
**B_D7**	0.09	0.04	0.09	0.00	0.06	0.00	0.09	0.00	0.00	0.00	0.00	0.00	0.00	0.02	0.00	-0.03
**B_D8**	0.11	0.03	0.00	0.03	0.01	0.00	0.00	0.01	0.00	0.00	-0.01	0.00	0.00	0.00	0.00	0.00
**M_D1**	0.01	0.02	0.02	0.00	0.01	0.00	0.00	0.00	0.02	0.01	0.00	0.00	0.06	0.00	0.06	0.03
**M_D2**	-0.02	0.00	0.00	0.00	-0.04	0.00	0.00	0.00	0.02	0.12	0.11	0.00	0.00	0.00	0.01	0.01
**M_D3**	0.00	0.00	0.02	0.00	0.04	0.01	0.00	0.00	0.05	0.05	0.36	0.03	0.03	0.13	0.05	0.00
**M_D4**	0.06	0.03	0.04	0.00	0.03	0.00	0.03	0.01	0.03	0.01	0.03	0.08	0.06	0.12	0.00	0.06
**M_D5**	0.00	0.00	0.00	0.00	0.00	0.00	0.00	0.00	0.07	0.02	0.00	0.05	0.20	0.02	0.00	0.09
**M_D6**	-0.02	0.00	0.00	0.00	0.00	0.00	0.00	0.00	0.02	0.01	0.03	0.07	0.00	0.05	0.02	0.01
**M_D7**	0.04	0.03	0.06	0.00	0.00	0.00	0.00	0.00	0.12	0.18	0.00	0.09	0.03	0.15	0.23	0.12
**M_D8**	0.00	0.00	0.00	0.00	0.00	0.03	0.00	0.00	0.07	0.09	0.00	0.00	0.00	0.01	0.08	0.22

Note. Independent variables (i.e., predictors) are in columns, and dependent variables are in rows. Participants' depressive symptoms were evaluated in eight items, including D1 depressiveness ("I felt depressed."), D2 effortfulness ("I felt everything I did was an effort."), D3 restlessness ("My sleep was restless."), D4 unhappiness ("I was happy.", reverse scored item), D5 loneliness ("I felt lonely."), D6 unenjoyment ("I enjoyed life.", reverse scored item), D7 sadness ("I felt sad."), and D8 felt life could not go on ("I felt my life could not go on."). Each item was self-reported on a 4-point scale, with score ranging from 1 = *rarely or none of the time* to 4 = *most or all of the time*.

Table S3. Edges matrix of the girl-father cross-lagged panel network.

	G_D1	G_D2	G_D3	G_D4	G_D5	G_D6	G_D7	G_D8	F_D1	F_D2	F_D3	F_D4	F_D5	F_D6	F_D7	F_D8
**G_D1**	0.02	0.04	0.00	0.00	0.00	0.00	0.00	0.00	0.08	0.04	0.00	0.00	0.00	0.00	0.00	0.01
**G_D2**	0.00	0.00	0.00	0.00	0.00	0.00	0.02	0.02	0.00	0.00	0.00	0.00	0.00	0.00	0.00	0.00
**G_D3**	0.00	0.00	0.16	0.00	0.04	0.00	0.00	0.00	0.00	0.00	0.00	0.00	0.00	0.03	0.00	0.00
**G_D4**	0.00	0.00	0.02	0.12	0.00	0.09	0.07	0.05	0.00	0.00	0.00	0.06	0.00	0.05	0.00	0.00
**G_D5**	0.12	0.02	0.04	0.12	0.14	0.06	0.05	0.01	0.00	0.00	0.00	0.04	0.00	0.10	0.00	0.00
**G_D6**	0.01	0.00	0.00	0.06	0.09	0.07	0.00	0.00	0.05	0.04	0.00	0.00	0.00	0.00	0.00	0.04
**G_D7**	0.12	0.09	0.00	0.00	0.09	0.00	0.13	0.07	0.00	0.00	0.00	0.00	0.00	0.00	0.00	0.00
**G_D8**	0.10	0.13	0.11	0.00	0.08	0.13	0.13	0.15	-0.06	0.00	0.00	0.01	0.00	0.00	0.00	0.00
**F_D1**	0.01	0.00	0.01	0.00	0.00	0.00	0.00	0.00	0.24	0.15	0.06	0.00	0.12	0.06	0.11	0.01
**F_D2**	0.00	0.00	0.00	-0.04	0.00	0.00	0.00	0.02	0.02	0.04	0.00	0.00	0.02	0.00	0.00	0.05
**F_D3**	0.03	0.00	0.05	0.00	0.00	0.00	0.00	0.02	0.05	0.05	0.33	0.06	0.09	0.11	0.00	0.00
**F_D4**	0.00	0.00	0.00	0.00	0.01	0.00	0.00	0.02	0.00	0.04	0.09	0.10	0.04	0.04	0.01	0.00
**F_D5**	0.00	0.00	0.00	0.04	0.00	0.03	0.01	0.00	0.00	0.00	0.05	0.00	0.22	0.00	0.00	0.00
**F_D6**	0.03	0.00	0.00	0.00	0.00	0.01	0.00	0.00	0.04	0.01	0.00	0.15	0.01	0.20	0.05	0.06
**F_D7**	0.00	0.00	0.00	0.00	0.00	0.00	0.00	0.00	0.00	0.11	0.02	0.00	0.00	0.00	0.10	0.04
**F_D8**	0.00	0.00	0.00	0.00	0.00	0.00	0.00	-0.03	0.09	0.00	0.01	-0.01	0.00	0.00	0.12	0.11

Note. Independent variables (i.e., predictors) are in columns, and dependent variables are in rows. Participants' depressive symptoms were evaluated in eight items, including D1 depressiveness ("I felt depressed."), D2 effortfulness ("I felt everything I did was an effort."), D3 restlessness ("My sleep was restless."), D4 unhappiness ("I was happy.", reverse scored item), D5 loneliness ("I felt lonely."), D6 unenjoyment ("I enjoyed life.", reverse scored item), D7 sadness ("I felt sad."), and D8 felt life could not go on ("I felt my life could not go on."). Each item was self-reported on a 4-point scale, with score ranging from 1 = *rarely or none of the time* to 4 = *most or all of the time*.

Table S4. Edges matrix of the girl-mother cross-lagged panel network.

	G_D1	G_D2	G_D3	G_D4	G_D5	G_D6	G_D7	G_D8	M_D1	M_D2	M_D3	M_D4	M_D5	M_D6	M_D7	M_D8
**G_D1**	0.07	0.08	0.00	0.00	0.00	0.00	0.01	0.03	-0.02	0.00	0.01	0.00	0.00	0.00	-0.06	0.00
**G_D2**	0.00	0.01	0.00	0.00	0.00	0.00	0.02	0.00	0.01	0.00	0.00	0.00	0.00	0.00	-0.06	-0.05
**G_D3**	0.00	0.00	0.16	0.00	0.00	0.00	0.00	0.00	-0.07	0.00	0.00	0.00	0.00	0.00	-0.01	0.02
**G_D4**	0.00	0.00	0.01	0.11	0.00	0.07	0.03	0.01	0.02	0.02	0.00	0.00	0.00	0.00	0.05	0.08
**G_D5**	0.16	0.04	0.05	0.05	0.14	0.07	0.09	0.00	0.00	0.02	0.03	0.00	0.01	0.00	0.07	0.05
**G_D6**	0.02	0.04	0.00	0.04	0.10	0.06	0.04	0.01	0.00	0.01	-0.02	0.00	0.00	0.00	-0.02	-0.04
**G_D7**	0.03	0.04	0.03	0.00	0.09	0.05	0.10	0.03	0.03	0.00	0.00	0.00	0.00	0.00	0.01	0.00
**G_D8**	0.16	0.15	0.08	0.00	0.06	0.02	0.14	0.16	0.04	0.00	0.03	0.03	0.00	0.00	0.09	0.00
**M_D1**	0.00	0.00	0.00	0.00	0.00	0.00	0.00	0.00	0.13	0.04	0.00	0.00	0.02	0.01	0.05	0.00
**M_D2**	0.00	0.00	0.00	0.00	0.02	0.00	0.00	0.00	0.07	0.19	0.05	0.03	0.04	0.00	0.06	0.07
**M_D3**	0.00	0.00	0.02	0.00	0.00	0.00	0.01	0.00	0.08	0.01	0.37	0.00	0.04	0.00	0.07	0.03
**M_D4**	-0.02	0.00	0.00	0.00	0.00	0.00	0.00	0.00	0.00	0.05	0.04	0.09	0.00	0.08	-0.05	0.00
**M_D5**	0.00	0.00	0.00	0.00	0.00	0.00	0.03	0.00	0.15	0.12	0.13	0.04	0.25	0.05	0.03	0.07
**M_D6**	0.00	0.00	0.02	0.00	0.00	0.00	0.00	0.00	0.06	0.00	0.00	0.10	0.01	0.12	0.05	0.02
**M_D7**	0.00	0.00	0.00	-0.03	0.00	0.00	0.00	0.00	0.08	0.04	0.00	0.09	0.00	0.11	0.31	0.06
**M_D8**	0.00	0.00	0.00	0.00	0.00	0.00	0.00	0.00	0.00	0.08	0.00	0.01	0.16	0.00	0.12	0.25

Note. Independent variables (i.e., predictors) are in columns, and dependent variables are in rows. Participants' depressive symptoms were evaluated in eight items, including D1 depressiveness ("I felt depressed."), D2 effortfulness ("I felt everything I did was an effort."), D3 restlessness ("My sleep was restless."), D4 unhappiness ("I was happy.", reverse scored item), D5 loneliness ("I felt lonely."), D6 unenjoyment ("I enjoyed life.", reverse scored item), D7 sadness ("I felt sad."), and D8 felt life could not go on ("I felt my life could not go on."). Each item was self-reported on a 4-point scale, with score ranging from 1 = *rarely or none of the time* to 4 = *most or all of the time*.
